# Transgenic Exosomes for Thymus Regeneration

**DOI:** 10.3389/fimmu.2019.00862

**Published:** 2019-04-24

**Authors:** Krisztina Banfai, Kitti Garai, David Ernszt, Judit E. Pongracz, Krisztian Kvell

**Affiliations:** ^1^Department of Pharmaceutical Biotechnology, Faculty of Pharmacy, University of Pécs, Pécs, Hungary; ^2^Szentagothai Research Center, University of Pécs, Pécs, Hungary; ^3^Faculty of Medicine, Institute of Physiology, University of Pécs, Pécs, Hungary

**Keywords:** aging, thymus, exosome, Wnt4, miR27b

## Abstract

During senescence, Wnt4 expression is down-regulated (unlike their Frizzled receptors), while PPARgamma expression increases in the thymus. Together, these changes allow for thymic degeneration to occur, observed as adipose involution. However, when restored, Wnt4 can efficiently counteract PPARgamma and prevent thymic senescence from developing. The Wnt-pathway activator miR27b has also been reported to inhibit PPARgamma. Our goal was to evaluate the Wnt4 and miR27b levels of Wnt4-transgenic thymic epithelial cell (TEC)-derived exosomes, show their regenerative potential against age-related thymic degeneration, and visualize their binding and distribution both *in vitro* and *in vivo*. First, transgenic exosomes were harvested from Wnt4 over-expressing TECs and analyzed by transmission electron microscopy. This unveiled exosomes ranging from 50 to 100 nm in size. Exosomal Wnt4 protein content was assayed by ELISA, while miR27b levels were measured by TaqMan qPCR, both showing elevated levels in transgenic exosomes relative to controls. Of note, kit-purified TEI (total exosome isolate) outperformed UC (ultracentrifugation)-purified exosomes in these parameters. In addition, a significant portion of exosomal Wnt4 proved to be displayed on exosomal surfaces. For functional studies, steroid (Dexamethasone or DX)-induced TECs were used as cellular aging models in which DX-triggered cellular aging was efficiently prevented by transgenic exosomes. Finally, DiI lipid-stained exosomes were applied on the mouse thymus sections and also iv-injected into mice, for *in vitro* binding and *in vivo* tracking, respectively. We have observed distinct staining patterns using DiI lipid-stained transgenic exosomes on sections of young and aging murine thymus samples. Moreover, *in vivo* injected DiI lipid-stained transgenic exosomes showed detectable homing to the thymus. Of note, Wnt4-transgenic exosome homing outperformed control (Wnt5a-transgenic) exosome homing. In summary, our findings indicate that exosomal Wnt4 and miR27b can efficiently counteract thymic adipose involution. Although extrapolation of mouse results to the human setting needs caution, our results appoint transgenic TEC exosomes as promising tools of immune rejuvenation and contribute to the characterization of the immune-modulatory effects of extracellular vesicles in the context of regenerative medicine.

## Introduction

Transcription factor FoxN1 is the mastermind of thymus organogenesis and identity (1), and is also an acknowledged direct molecular target of the glycolipoprotein Wnt4 (2). As a consequence, Wnt4 plays a key role during embryonic thymus development (3, 4) and the maintenance of its identity in adulthood (5–7). Thymic epithelial cells secrete less Wnt4, while their Frizzled receptors (Fz4 and Fz6) become up-regulated indicating a potential compensatory mechanism and possibly enhanced Wnt4-binding (8). This loss of Wnt4 expression weakens thymic epithelial identity and allows for thymic adipose involution to occur (9). This latter process leads to the expansion of thymic adipose tissue orchestrated by transcription factor PPARgamma (10). The Wnt/b-catenin pathway and PPARgamma have been reported to act as mutual inhibitors of one another in several tissue contexts, including the thymus (11–13). We have previously shown that the addition of exogenous Wnt4 reinforces thymic epithelial identity and confers resistance in a steroid-induced model of senescence through suppressing PPARgamma (2, 14).

Previous records reported that Wnt4 loses its activity when purified as a sole compound, but retains activity as supernatant fraction (15). In harmony, recent publications of various tissue contexts have suggested that Wnt molecules (including Wnt4) travel in conjunction with extracellular vesicles (EVs), more specifically exosomes (12, 16). It has also been reported that a significant portion of the Wnts—including Wnt4—may actually be displayed on exosomal surfaces. Along with Wnt4, the Wnt-pathway activator miR27b has also been shown to specifically inhibit PPARgamma activity via binding to its promoter region (17, 18). Similar to the Wnts above, miRNA species have also been suggested to preferentially reside and travel in EVs, especially in exosomes (19, 20).

EVs are released by most cell types of all phyla and mediate various biological effects. EVs are classified by their size where exosomes represent the smallest vesicles with a diameter of between 30 and 200 nm. Exosomes are produced by a multi-step process where multi-vesicular bodies (MVBs) are formed first by cell-membrane invaginations, followed by the extracellular release of exosomes (21). Biological functions attributed with exosomes encompass several physiological and pathological conditions, including cell and tissue regeneration (22, 23). The thymus epithelium has also been reported to be a rich source of exosomes with key immunological relevance (24–26) e.g., in thymocyte selection (25, 27), Yet to date, TEC (thymic epithelial cell) exosomes have not been linked with thymus tissue regeneration. Thymus tissue regeneration is not only relevant for aging studies, but also in conditions that accelerate thymus degeneration due to environmental stimuli including specific toxins, viruses, or heavy metals (28).Due to the reasons above, we have set out to characterize transgenic exosomes produced by Wnt4 over-expressing TECs for their Wnt4 and miR27b content, to test their biological activity in the context of tissue regeneration, and distribution properties both *in vitro* and *in vivo*.

## Materials and Methods

### Cell Cultures

*In vitro* experiments were performed using the TEP1 primary-derived (BALB/c) thymic epithelial cell lines or the A549 human lung epithelial cell line (A549 served as control producer cell compared to TEP1). The Wnt4 over-expressing version of TEP1 and the Wnt5a over-expressing version of A549 were generated via lentiviral transfection linked to the green fluorescent protein (GFP) as published previously (Wnt5a served as control compared to Wnt4) (29, 30). Cells were maintained in DMEM (Dulbecco's Modified Eagle's medium, Lonza) supplemented with 10% FBS (EuroClone), Penicillin-Streptomycin, L-glutamine, Hepes buffer, non-essential amino acids (Lonza), and β-mercapto-ethanol (Sigma). In order to differentiate thymic epithelial cells (TECs) toward adipose, lineage steroid treatment was used. Dexamethasone (DX) was diluted from a stock solution of 4 mg/mL to a final concentration of 1 μM as formerly described (10). To counteract the aging effect of steroid treatment, isolated Wnt4 exosomes were added to the cell cultures.

### Flow-Cytometry

Cell suspensions were prepared from both TEP1 and Wnt4 over-expressing TEP1 cell lines in order to check the presence of Wnt4 over-expression via analyzing the GFP-positive cells. A total of 150,000 cells were collected and washed with 1x PBS (Fisher BioReagents) then fixed using paraformaldehyde containing PBS solution. BD FACSCanto™ II flow-cytometer (Becton Dickinson) was used for data acquisition at a medium flow rate and stopped at 10,000 events. Measurements were performed and analyzed with BD FACSDiva Software version 6.1.3.

### Exosome Staining, Collection and Isolation

Control mouse TECs(thymic epithelial cells), Wnt4 over-expressing mouse TECs and Wnt5a over-expressing human A549 cells were cultured in Stemline® T Cell Expansion Medium (Merck) and serum-free DMEM (Lonza) until they reached 80–90% confluence. Serum-free media were used to eliminate the effect of serum-derived exosomes. Equal volume of FBS-free cell culture media were collected from T75 tissue culture flasks (TPP) and centrifuged at 2,000 g for 30 min to completely remove cell debris and apoptotic bodies. Supernatants were filtered through a 0.45 μm filter (Merck Millipore) and incubated overnight at 4°C having added Total Exosome Isolation Reagent (Invitrogen). Following a 1 h centrifugation at 10,000 g, pellets were collected and re-suspended in sterile PBS (GE Healthcare Life Sciences) for further use. Exosomes were fluorescently-stained using DiI lipid stain (Invitrogen). DiI lipid-stain was added to the cell culture medium the day before collecting cell supernatant. DiI lipid-stain stock solution (50 mg/mL dissolved in DMSO) was diluted 10,000-fold (31).

### Transmission Electron Microscopy

Pelleted exosomes were added in 50 μl of PBS onto mesh grids and dried overnight without the use of any fixative (32). Contrast staining was performed using uranyl-acetate and lead-citrate. Exosomes were examined using a Morgagni 268D transmission electron microscope. Images were acquired using an integrated MegaView III digital camera (Olympus Soft Imaging Solutions GmbH).

### Immune-Fluorescent Staining

Immune-fluorescent staining was performed on 2-month-old and 21-month-old mouse thymus cryosections. Seven micrometer thick tissue sections were mounted onto glass slides and dried overnight. Tissue samples were fixed with cold acetone then unspecific protein-protein interactions were blocked with 5% BSA in PBS solution before applying fluorochrome-conjugated primary antibodies. FITC-conjugated a-mouse CD326 (EpCAM) (Clone G8.8, BioLegend) was used at 1:100 dilution and DAPI (1:1,000, Life Technologies) was added as a nuclear counterstain. Slides were also incubated overnight with previously DiI-stained Wnt4 exosomes. Following washing steps with 1x PBS, samples were imaged using Nikon Eclipse Ti-U microscope equipped with a CCD camera (Andor 4Zyla 5.5) and images were captured using NIS-Elements Software. Images were analyzed using ImageJ Software. Thymus lobes of iv-injected mice were sectioned to 5 μm thickness and the same staining procedure was used as described above. Staining procedures were optimized using previous literature data (33–36).

### RNA Isolation, cDNA Preparation, qRT-PCR, TaqMan Array

**Figure 1:**
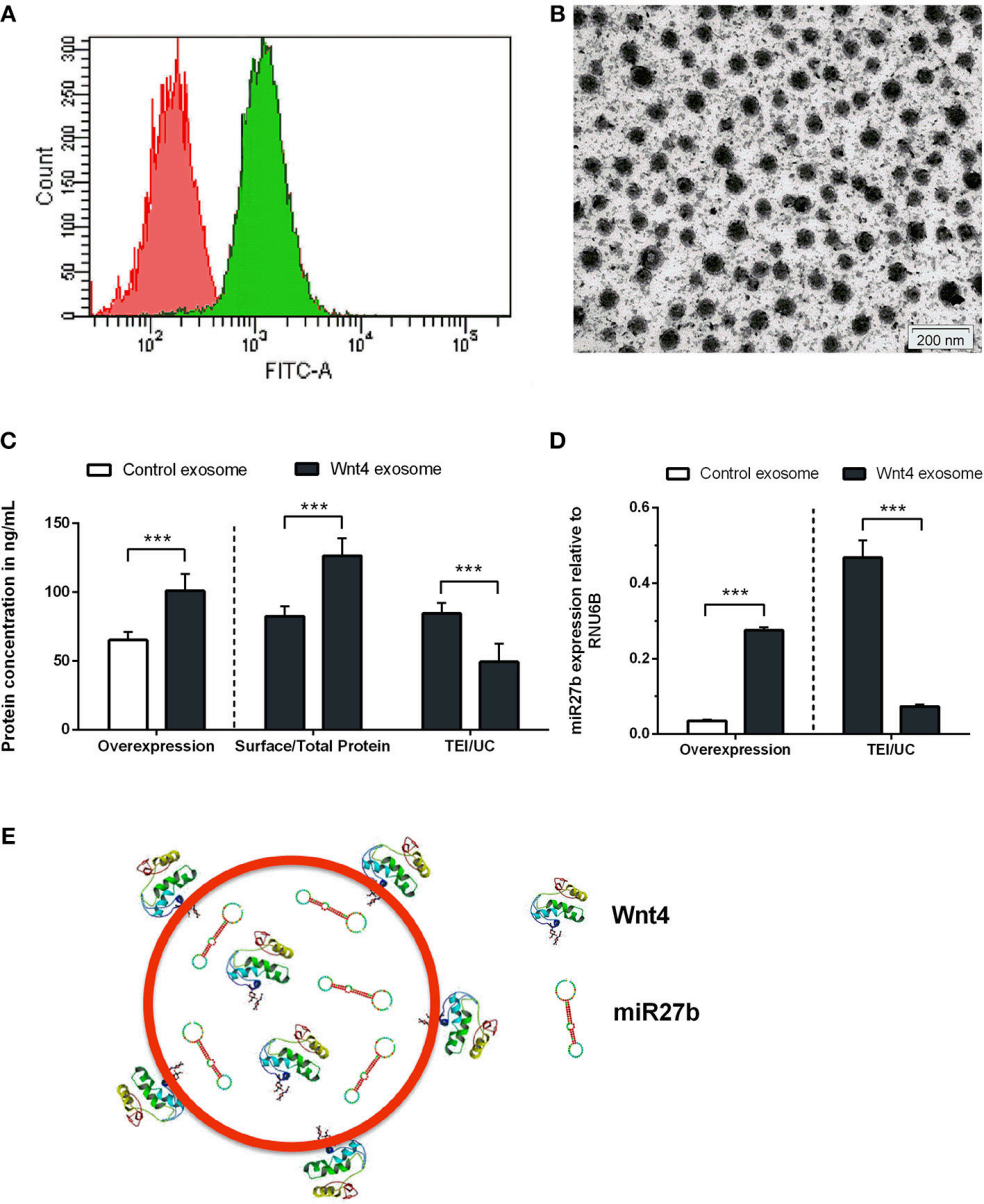
Characterization of transgenic exosomes. Flow-cytometric analysis of control (shown in red) and GFP+ Wnt4 over-expressing TECs (thymic epithelial cells) (shown in green) is presented **(A)**. FITC-A corresponds to FL1 channel where GFP-emitted fluorescence is detected. Please note that FITC-A scale is logarithmic. Transmission electron micrograph shows TEI exosomes of approx. 50–100 nm in size **(B)**. Wnt4 protein **(C)** and miR27b RNA **(D)** levels of TEI (total exosome isolate) and UC (ultracentrifuged) exosomes are shown as obtained by ELISA and TaqMan qPCR, respectively. From left to right column pairs show level of over-expression, surface/total content, and TEI/UC content, as applicable. Absolute concentration is shown in ng/ml for Wnt4 **(C)** and absolute copy number is shown for miR27b relative to rnu6b **(D)**. Eight **(C)** and three **(D)** replicates were used for statistical analysis. Significant differences are shown by asterisks (n/a for not applicable, ns for not significant, *p ≤ 0.05, **p ≤ 0.01, ***p ≤ 0.001) and were calculated using independent samples *t*-test. For exact numerical values and statistical analysis please refer to Supplementary Material. Representative drawing of a DiI lipid-stained (red) Wnt4-transgenic exosome showing Wnt4 (surface and internal) content and miR27b (internal) cargo **(E)**.

**Figure 2:**
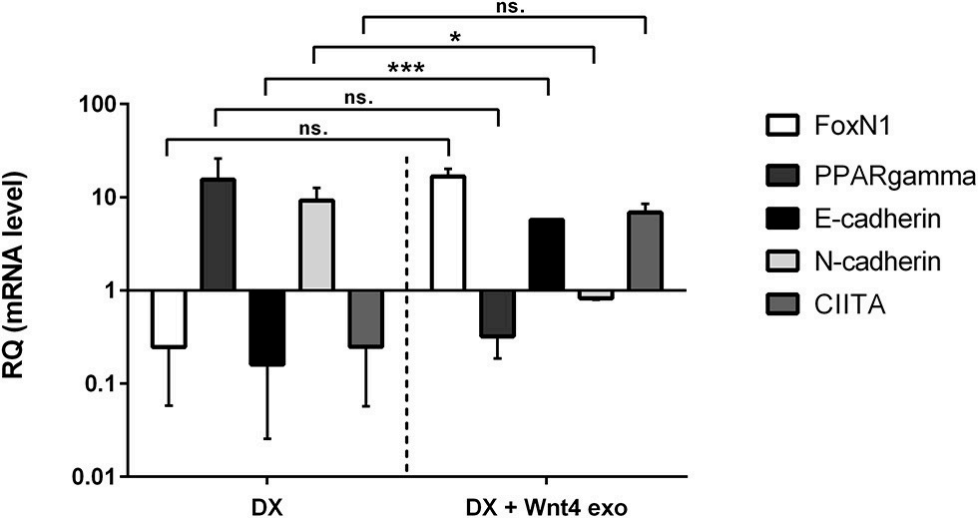
*In vitro* biological effect of transgenic exosomes. Gene expression changes of FoxN1, PPARgamma, E-cadherin, N-cadherin, and CIITA are shown as measured by SYBR-green qPCR following steroid (DX)-treatment alone (DX, left half of figure), or in combination with transgenic exosomes (DX + Wnt4 exo, right half of figure). Three samples of each were used for statistical analysis. Fold change (relative quantity or RQ) expression is shown, where RQ = 1 represents baseline control. RQ is relative to control level (left half of figure), or relative to DX-treatment (right half of figure). Please note that Y-axis is logarithmic. Significant differences are shown by asterisks (n/a for not applicable, ns for not significant, *p ≤ 0.05, **p ≤ 0.01, ***p ≤ 0.001) and were calculated using independent samples *t*-test. For exact numerical values and statistical analysis please refer to Supplementary Material.

Total RNA was isolated using NucleoSpin RNA II Kit (Macherey-Nagel). High Capacity cDNA Reverse Transcription Kit (Applied Biosystems) was used for preparation of cDNA to a final concentration of 1 μg/μL. For qPCR analysis, PikoReal™ Real-Time PCR System (Thermo Fisher Scientific) was used adding Luminaris Color HiGreen qPCR Master Mix (Thermo Fisher Scientific) to the samples. Gene expression was normalized to mouse β-actin and HPRT housekeeping genes (See Figures 1, 2 for detailed primer list). To detect miR27b levels in exosomes, Total Exosome RNA & Protein Isolation Kit (Invitrogen) was used for miRNA isolation. cDNA synthesis was carried out using High Capacity cDNA Reverse Transcription Kit (Applied Biosystems) and specific primers for U6B as endogenous control and miR27b as target gene. Quantification of miR27b was performed using specific gene targeted TaqMan™ MicroRNA Assay adding TaqMan Universal PCR Master Mix (Applied Biosystems). Measurements were run on a PikoReal™ Real-Time PCR System as well using FAM as a fluorophore. Data evaluation was accomplished using Microsoft Excel.

### ELISA

Human Wnt4 ELISA Kit (Merck) was used to measure the Wnt4 protein levels of isolated exosomes. Exosomes isolated from TEP1 cell culture media were used as controls. We aimed to quantify surface and total exosome protein levels separately. To detect surface proteins, pelleted exosomes were dissolved in PBS (for intact exosomes), while to determine total protein concentration, exosomes were diluted in Exosome Resuspension Buffer (for disintegrated exosomes) as suggested by Total Exosome RNA & Protein Isolation Kit Manual (Invitrogen). Plates were measured immediately at 450 nm using EnSpire® Multimode Plate Reader (PerkinElmer) with its integrated data analysis software. Using OD values mean absorbance of standards and samples were calculated and results were analyzed with the help of Microsoft Excel. Standard curve was plotted and protein concentrations were calculated in ng/ml. The obtained *R*^2^ values showed >0.91 alignment to the standard curve in all cases.

### Ultracentrifugation

In order to compare TEI efficiency (37) with standard ultracentrifugation methods confirming previous literature data (38, 39), 1 ml serum-free medium containing exosomes was centrifuged at 100,000 g for 3 h at 4°C (40) using Sorvall™ MTX 150 Micro-Ultracentrifuge (Thermo Scientific™). Pelleted exosomes were re-suspended in PBS and used for further experiments. Protein concentration of ultracentrifuged exosomes were determined with ELISA and following miRNA isolation, miR27b levels were assessed by TaqMan™ MicroRNA Assay.

### *In vivo* Exosome Homing

Eight week-old BALB/c mice were used for intravenous introduction of DiI lipid-stained exosomes in a pilot study (41, 42). For control purpose a mouse received 2 doses of DiI lipid-stained Wnt5a exosomes diluted in 200 μL of sterile PBS. Another mouse was injected with the same dosage of DiI lipid-stained Wnt4 exosomes diluted in 200 μL of sterile PBS. After 24 h, mice were sacrificed and organs were analyzed for fluorescent intensity with the help of IVIS Lumina III *in vivo* Imaging System. Imaging of the thymus, lungs, liver, and spleen was performed at 520 nm using Living Image Software. Thymus lobes were embedded into cryomold and sectioned to confirm tissue homing. Mice were housed under minimal disease (MD) conditions and kept in the Laboratory Animal Core Facility of the University. Experimental procedures were carried out according to the “1988/XXVIII act of the Hungarian Parliament on Animal Protection (243/1988)” which complies with recommendations of the Helsinki Declaration. All animal experiments were performed with the consent of the Ethics Committee on Animal Research of the University (ref. no.: #BA02/2000-46/2016).

### Statistical Analysis

Statistical analyses were conducted using SPSS version 22. Descriptive statistics (mean ± SD) were calculated for all data. Normality was assessed using the Shapiro-Wilk test (*n* < 50). Comparisons were performed using one sample *t*-test and independent samples *t*-test. The level of significance was set at *P* ≤ 0.05.

## Results

### Characterization of Transgenic Exosomes

Stable Wnt over-expressing BALB/c TEC (thymic epithelial cell) lines have been generated using lentiviral vectors as described previously (30). In the current setting, we focused on the characterization of exosomes secreted by the transgenic cell lines. Purity and transgenic status of cell lines was confirmed by GFP expression provided by the same bicistronic construct that also drives Wnt secretion. Flow-cytometric analysis verified that the transgenic cells express GFP and hence secrete Wnt4 at a uniform level (see Figure 1A). TECs have been reported to be rich EV and exosome sources. Using a commercially available PEG-based kit (40) we then enriched transgenic exosomes (TEI: total exosome isolate) from TEC line supernatant. The enriched supernatant fraction was confirmed by transmission electron microscopy to contain of uniform-sized exosomes of approx. 50–100 nm in diameter (see Figure 1B). Along with Wnt4, the Wnt-pathway activator miR27b has also been reported to suppress PPARgamma (17, 18, 43, 44). For this reason, we have measured both Wnt4 protein and miR27b RNA quantities in transgenic exosomes relative to their control counterparts, using ELISA and TaqMan qPCR methods, respectively. As expected, both Wnt4 and miR27b showed elevated and statistically significant levels in exosomes of transgenic TECs compared to their control counterparts (see Figures 1C,D). Furthermore, we have measured total Wnt4 concentration (via lysis of exosomes) and surface Wnt4 concentration (using intact exosomes) showing that a significant portion of Wnt4 is surface-displayed, in harmony with literature (8, 19) (Figure 1C). Of note, UC (ultracentrifugation) has repeatedly provided poor exosomal protein and miRNA yield as compared to TEI (see Figures 1C,D). Figure 1E shows schematic drawing of DiI lipid-stained transgenic exosome with Wnt4 content and miR27b cargo.

### Biological Effect of Transgenic Exosomes

We have previously reported that the supernatant of the Wnt4 over-expressing BALB/c TEC (thymic epithelial cell) line confers resistance to steroid (Dexamethasone or DX)-induced accelerated aging in our cellular model system (2). In short, DX-treatment triggers adipose transformation of TECs via epithelial-to-mesenchymal transition (EMT) (10). We have used the same experimental setting using enriched transgenic exosomes instead of transgenic supernatant. As expected and shown by SYBR-green qPCR (see Figure 2), the applied DX-treatment triggers loss of TEC identity (decreased FoxN1 and MHCII or CIITA expression), promotes EMT (cadherin switch or decreased E-cadherin and increased N-cadherin expression), and initiates adipose transformation (increased PPARgamma expression). In perfect conformity with our previous reports using transgenic supernatant (2, 4), the transgenic exosomes also efficiently counteract all of the above gene expressional changes, preserving TEC identity, blocking EMT, and adipose transformation from developing.

### *In vitro* Binding and Distribution of Transgenic Exosomes

**Figure 3:**
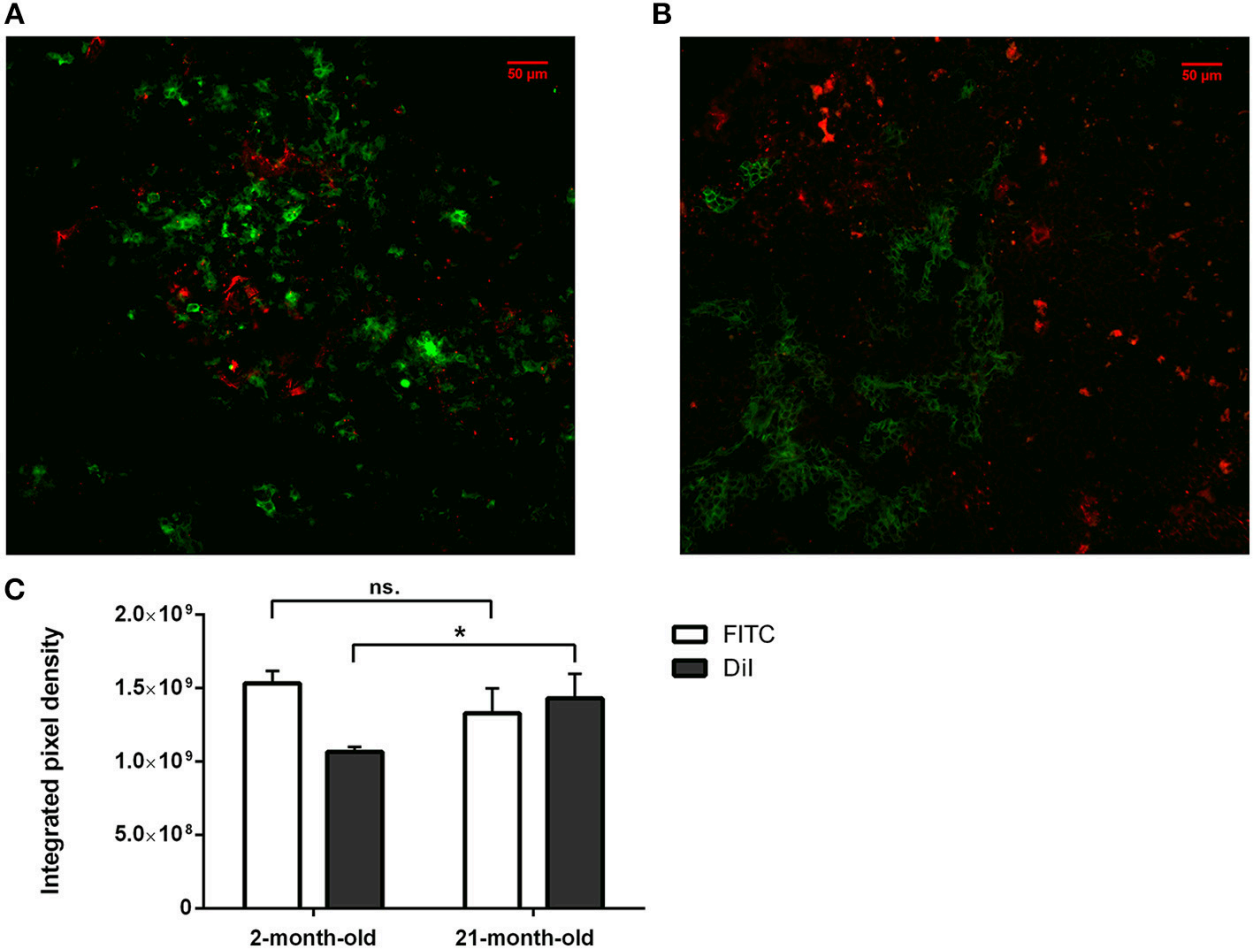
*In vitro* binding and distribution of transgenic exosomes. Frozen mouse thymic sections from young adult (2 months, **A**) and senior adult (21 months, **B**) are shown. Sections were labeled using EpCAM1-FITC (shown in green). Transgenic exosomes were pre-stained using DiI lipid stain (shown in red). Representative slide is presented. Data were calculated using five slides each. Integrated pixel density values are shown for EpCAM1 (shown in green) and DiI lipid stain (shown in red) **(C)**. Significant differences are shown by asterisks (n/a for not applicable, ns for not significant, *p ≤ 0.05, **p ≤ 0.01, ***p ≤ 0.001) as obtained using independent sample *t*-test. Data were calculated using five slides each. For exact numerical values and standard deviation please refer to Supplementary Material.

We have shown above that our transgenic exosomes harbor elevated amounts of Wnt4 of which a significant portion is surface-displayed. Previously we have reported that Wnt4-binding Frizzled receptors (Fz4 and Fz6) are up-regulated in aged TECs (thymic epithelial cells) as compared to young TECs (8). Next, we have tested whether thymic histological sections can bind transgenic exosomes using standard immune-fluorescent staining protocol. We were also interested to see if histological *in vitro* binding shows a particular pattern and furthermore, if this pattern changes with the age of the epithelium as suggested by Frizzled up-regulation (2, 8). For this reason, DiI lipid-stained transgenic exosomes have been applied on thymic sections of mice of 2-month-old (young) and 21-month-old (aged) for histological staining. As anticipated, both young and old mouse thymic sections efficiently bind transgenic exosomes (see Figures 3A,B, respectively). Relative to the histological presentation of the EpCAM-1^++^ medullary epithelium, the transgenic exosomes showed preferential binding to medullary regions at a young age (see Figure 3A), while a more profound cortical binding pattern was observed at an old age (see Figure 3B). Not only does the binding pattern change with age (shifting from medullary at a young age to cortical at an old age), but binding efficiency also increases with age (observed as increased, statistically significant DiI/fluorescent integrated pixel density at old age compared to young age, see Figure 3C). Please also note that the medullary area shrinks with age relative to other areas, in harmony with our previous records (4) observed here as mild indicative decrease of FITC integrated pixel density at old age compared to young age (see Figure 3C).

### *In vivo* Binding and Distribution of Transgenic Exosomes

**Figure 4:**
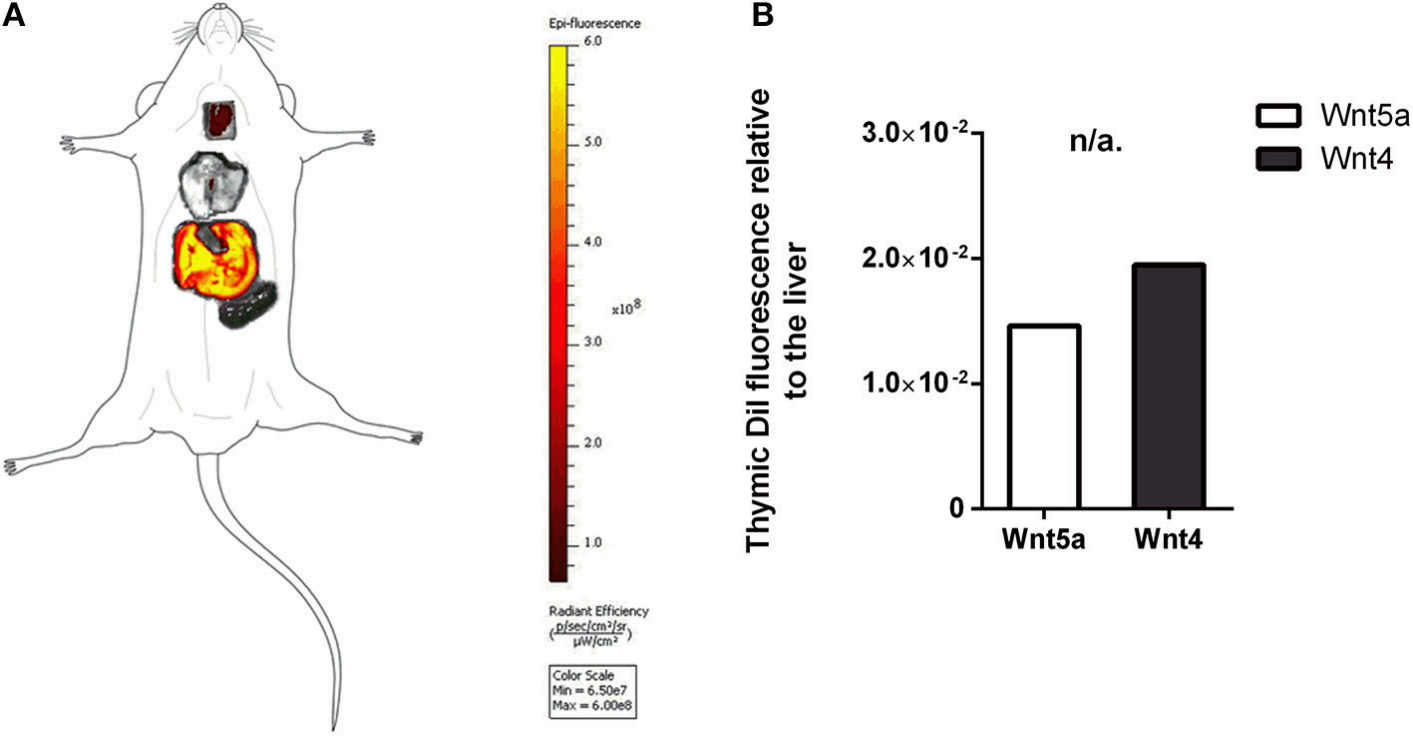
*In vivo* binding and distribution of transgenic exosomes. DiI lipid-stained and iv-injected exosomes show differential topological distribution patterns (radiant efficiency) after 24 h. Organotopic distribution was reconstructed using standard mouse contour. The thymus, lungs, spleen, and liver were analyzed using IVIS Lumina imaging **(A)**. Radiant efficiency of thymus relative to liver (RQ) is shown for of DiI lipid-stained and iv-injected transgenic exosomes after 24 h for Wnt5a (control) and Wnt4 (sample) **(B)**. Significant differences are shown by asterisks (n/a for not applicable, ns for not significant, *p ≤ 0.05, **p ≤ 0.01, ***p ≤ 0.001) as obtained using independent samples *t*-test. Pilot study is shown. For exact numerical values please refer to Supplementary Material.

**Figure 5:**
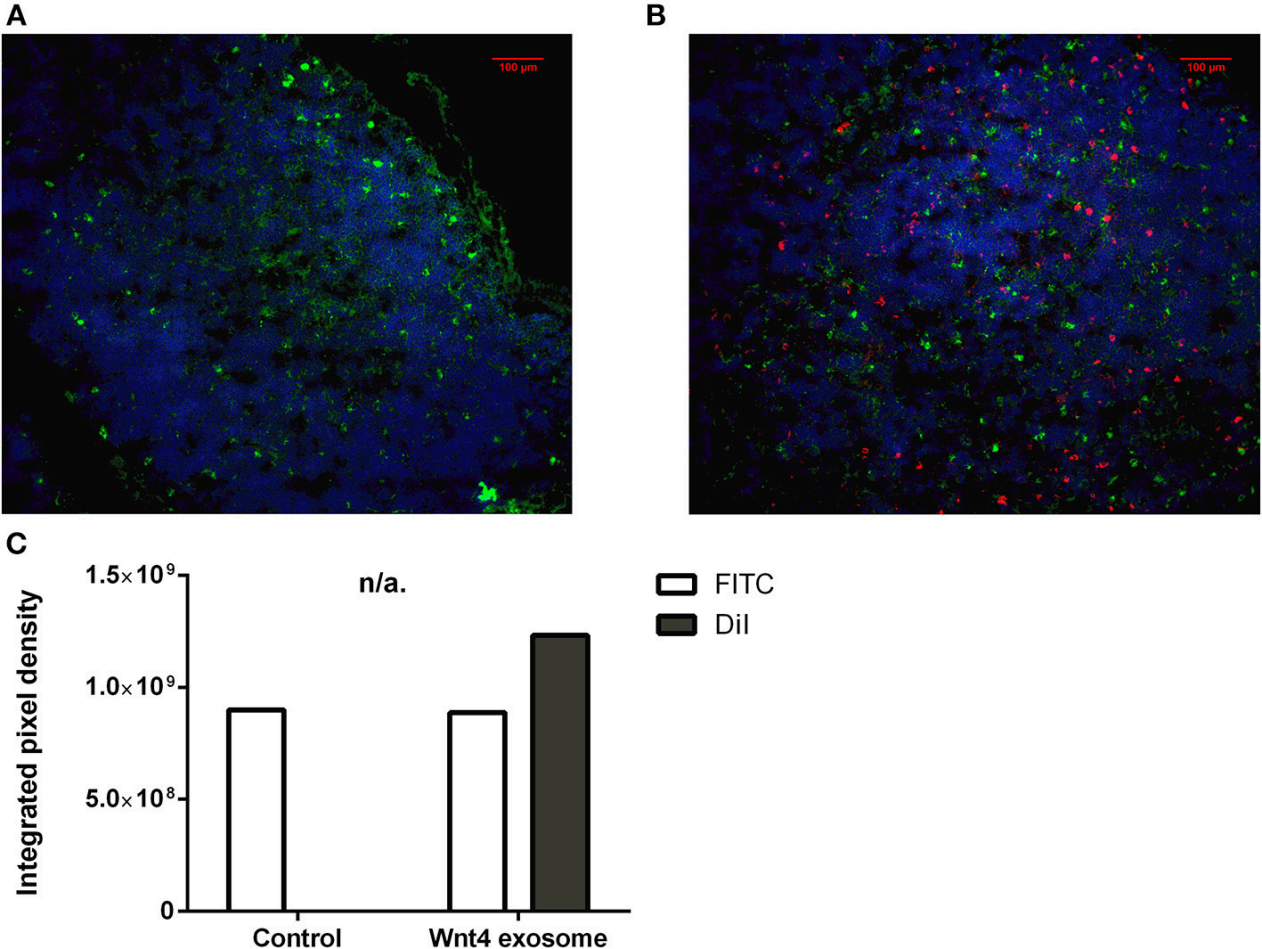
*Ex vivo* binding and distribution of transgenic exosomes. Frozen mouse thymic sections from control **(A)** and DiI lipid-stained Wnt4-transgenic exosome iv-injected mouse **(B)** are shown 24 h after administration. Sections were stained for EpCAM1-FITC (shown in green). Transgenic exosomes were pre-stained using DiI lipid stain (shown in red). DAPI was used as nuclear counter-stain (shown in blue). Integrated pixel density values are shown for EpCAM1 (shown in green) and DiI lipid stain (shown in red) **(C)**. Significant differences are shown by asterisks (n/a for not applicable, ns for not significant, *p ≤ 0.05, **p ≤ 0.01, ***p ≤ 0.001) as obtained using independent samples *t*-test. Pilot study is shown. For exact numerical values please refer to Supplementary Material.

The thymic epithelium has been reported to be highly active in exosome trafficking (24, 25, 45). We have shown above that our Wnt4-transgenic exosomes readily bind to the thymic epithelium *in vitro* on histological sections. However, we were interested to see if *in vivo* binding of transgenic exosomes also occurs in the thymic epithelium. For this reason, DiI lipid-stained Wnt5a-transgenic (serving as control) human (A549), and Wn4-transgenic (serving as sample) BALB/c (TEP1) exosomes have been injected into tail veins of young adult BALB/c mice to check their *in vivo* topological distribution after 24 h. It has been reported that certain organs (e.g., liver, lungs, and spleen) capture a significant portion of exosomes rapidly and non-specifically following systemic administration (46). Using IVIS Lumina III imaging and performing topological reconstruction of murine organs over standard mouse contour, we were able to record detectable homing of transgenic exosomes to the thymus despite significant non-specific capture by other organs (e.g., liver, lungs, and spleen) (see Figure 4A). Fluorescent signals of DiI lipid-stained sample Wnt4-transgenic TEC (thymic epithelial cell) exosomes exceeded control values of Wnt5a-transgenic exosomes (see Figure 4B). It is worthy of note that thymic signals exceed pulmonary and splenic signals, despite significant size differences. Furthermore, immune-fluorescently labeled sections of the *in vivo* Wnt4-transgenic exosome-infiltrated thymus revealed the histological level homing pattern observed after 24 h (see Figures 5A,B) that allows for the quantitative evaluation of transgenic exosome homing (see Figure 5C).

## Discussion

In harmony with literature data, our results confirm that the thymic epithelium is a particularly rich source of EVs and exosomes (24, 25, 45). The Wnt4 over-expressing transgenic TEC (thymic epithelial cell) line proves to be a reliable source of transgenic exosomes that are easy to visualize, enrich to high purity, characterize, and apply in experiments. As anticipated, the transgenic exosomes contain elevated levels of Wnt4 protein, as well as the Wnt-pathway activator miR27b, which potentially synergizes with Wnt4 to counteract PPARgamma.

Based on our current results obtained with our thymus aging cellular model system the transgenic exosomes transmit the biological activity that was previously attributed to supernatant transfer (2, 4). The applied transgenic exosomes efficiently prevent steroid-triggered adipose transformation as supported by reinforced epithelial identity (increased FoxN1 and CIITA expression), lack of EMT (sustained E-cadherin and low N-cadherin expression) and resistance to adipose differentiation (low PPARgamma expression) in line with our previous reports using transgenic supernatants.

Histological level analysis of *in vitro* transgenic exosome binding provides information in more depth. At a young age (2-month-), transgenic exosomes readily bind to the thymic epithelium with slight medullary preference. In contrast, at an old age (21-month-), transgenic exosomes show a moderate cortical preference. Furthermore, transgenic exosomes show significantly higher overall binding frequency at an old age, suggesting that the senescent thymic epithelium readily adsorbs Wnt4-transgenic exosomes. This is supported by literature data and our own results showing that a significant portion of exosomal Wnt4 is surface-displayed and that Wnt4-binding Frizzled receptors (Fz4 and Fz6) are up-regulated in senescent TECs (8). Combined, our previous and current results suggest that as Wnt4 secretion decreases with age and since cortical areas show preferential binding of the remaining Wnt4 (a key factor of TEC identity) at an old age, age-related medullary involution may precede cortical involution due to the medullary lack of Wnt4-effect (10, 14, 47). To our knowledge, this is a novel molecular level explanation of accelerated medullary involution. Highlighting the relevance of our *in vitro* binding assay, our *in vivo* transgenic exosome homing assay shows detectable homing to the thymus when using mouse TEC-derived Wnt4-transgenic exosomes as opposed to control exosomes (human A549-derived Wnt5a-transgenic exosomes). Histological analysis of Wnt4-transgenic exosome-infiltrated thymus sections confirms their presence also showing distribution pattern and binding frequency.

In summary, based on our cellular aging model system, along with *in vitro* and *in vivo* binding and homing studies, respectively; our results confirm that transgenic exosomes readily and efficiently provide a permissive niche for immune regeneration in the mouse setting and hold promise for human application as well.

## Ethics Statement

Experimental procedures were carried out according to the 1988/XXVIII act of the Hungarian Parliament on Animal Protection (243/1988) which complies with recommendations of the Helsinki Declaration. All animal experiments were performed with the consent of the Ethics Committee on Animal Research of the University (ref. no.: #BA02/2000-46/2016).

## Author Contributions

KB performed histological, cellular- and molecular biology work in the project and was involved in manuscript preparation. KG prepared exosome fractions and performed statistical analysis. DE executed *in vivo* tracking. JP was involved in planning experiments, manuscript preparation and also supervised the host department. KK planned experiments, prepared the manuscript and supervised the project.

### Conflict of Interest Statement

The authors declare that the research was conducted in the absence of any commercial or financial relationships that could be construed as a potential conflict of interest.
