# Supplementary material for: Transgenic Exosomes for Thymus Regeneration
Source: Front Immunol. 2019 Apr 24;10:862. doi: 10.3389/fimmu.2019.00862 (PMC6499203; doi:10.3389/fimmu.2019.00862)
Supplement: Supplementary file 1 [file Table_1.DOCX]

**SUPPLEMENTARY MATERIAL**

**Numerical values and statistical analyses**

**Figure 1A.** TEC flow-cytometric analysis values

| **Cell lines** | **#Events** | **% Parent** | **FITC-A Mean** |
| --- | --- | --- | --- |
| Ctrl | 6747 | 43.2 | 176 |
| Wnt4 | 8187 | 85.8 | 1492 |

**Figure 1C.** Wnt4 protein levels of enriched exosomes

| **Control exosome** (n=8) | | | **Wnt4 exosome** (n=8) | | |
| --- | --- | --- | --- | --- | --- |
| **Mean** | **SD** | **Std. Error Mean** | **Mean** | **SD** | **Std. Error Mean** |
| 65.206 | 5.804 | 2.052 | 100.940 | 12.131 | 4.289 |

| \| Tests of Normality \| \| \| \| \| \| \| \| --- \| --- \| --- \| --- \| --- \| --- \| --- \| \|  \| Kolmogorov-Smirnov^a^ \| \| \| Shapiro-Wilk \| \| \| \| Statistic \| df \| Sig. \| Statistic \| df \| Sig. \| \| **Ctrl exosomes** \| 0.370 \| 3 \| 0.000 \| 0.786 \| 3 \| 0.082 \| \| **Wnt4 exosomes** \| 0.283 \| 3 \| 0.000 \| 0.934 \| 3 \| 0.505 \|   a. Lilliefors Significance Correction   \| Independent Samples Test \| \| \| \| \| \| --- \| --- \| --- \| --- \| --- \| \|  \| \| Levene's Test for Equality of Variances \| \| t-test for Equality of Means \| \| \| F \| Sig. \| t \| \| \| \|  \| Equal variances assumed \| 1.229 \| **0.286** \| -7.515 \| \| \| Equal variances not assumed \|  \|  \| -7.515 \| \|  \|  \| \| \| \| \| \| --- \| --- \| --- \| --- \| --- \| \|  \| \| t-test for Equality of Means \| \| \| \| df \| Sig. (2-tailed) \| Mean Difference \| \| \|  \| Equal variances assumed \| 14 \| **0.000** \| -35.733945 \| \| Equal variances not assumed \| 10.045 \| 0.000 \| -35.733945 \| |
| --- | --- | --- | --- | --- | --- | --- | --- | --- | --- | --- | --- | --- | --- | --- | --- | --- | --- | --- | --- | --- | --- | --- | --- | --- | --- | --- | --- | --- | --- | --- | --- | --- | --- | --- | --- | --- | --- | --- | --- | --- | --- | --- | --- | --- | --- | --- | --- | --- | --- | --- | --- | --- | --- | --- | --- | --- | --- | --- | --- | --- | --- | --- | --- | --- | --- | --- | --- | --- | --- | --- | --- | --- | --- | --- | --- | --- | --- | --- | --- | --- | --- | --- |

|  | | | |
| --- | --- | --- | --- |
|  | | t-test for Equality of Means | |
|  |  | Std. Error Difference | 95% Confidence Interval of the Difference |
|  |  |  | Lower |
|  | Equal variances assumed | 4.754784 | -45.931943 |
|  | Equal variances not assumed | 4.754784 | -46.321796 |

|  | | |
| --- | --- | --- |
|  | | t-test for Equality of Means |
|  |  | 95% Confidence Interval of the Difference |
|  |  | Upper |
|  | Equal variances assumed | -25.535946 |
|  | Equal variances not assumed | -25.146094 |

| **Surface Wnt4 protein content** (n=8) | | | **Total Wnt4 protein content** (n=8) | | |
| --- | --- | --- | --- | --- | --- |
| **Mean** | **SD** | **Std. Error Mean** | **Mean** | **SD** | **Std. Error Mean** |
| 82.439 | 7.336 | 2.594 | 126.424 | 12.701 | 4.491 |

| Tests of Normality | | | | | | |
| --- | --- | --- | --- | --- | --- | --- |
|  | Kolmogorov-Smirnov^a^ | | | Shapiro-Wilk | | |
|  | Statistic | df | Sig. | Statistic | df | Sig. |
| **Surface protein** | 0.286 | 3 | 0.000 | 0.930 | 3 | 0.490 |
| **Total protein** | 0.327 | 3 | 0.000 | 0.872 | 3 | 0.301 |

a. Lilliefors Significance Correction

| Independent Samples Test | | | | |
| --- | --- | --- | --- | --- |
|  | | Levene's Test for Equality of Variances | | t-test for Equality of Means |
|  |  | F | Sig. | t |
|  |  |  |  |  |
|  | Equal variances assumed | 4.816 | **0.046** | -8.482 |
|  | Equal variances not assumed |  |  | -8.482 |

|  | | | | |
| --- | --- | --- | --- | --- |
|  | | t-test for Equality of Means | | |
|  |  | df | Sig. (2-tailed) | Mean Difference |
|  |  |  |  |  |
|  | Equal variances assumed | 14 | 0.000 | -43.985656 |
|  | Equal variances not assumed | 11.202 | **0.000** | -43.985656 |

|  | | | |
| --- | --- | --- | --- |
|  | | t-test for Equality of Means | |
|  |  | Std. Error Difference | 95% Confidence Interval of the Difference |
|  |  |  | Lower |
|  | Equal variances assumed | 5.185697 | -55.107871 |
|  | Equal variances not assumed | 5.185697 | -55.374208 |

|  | | |
| --- | --- | --- |
|  | | t-test for Equality of Means |
|  |  | 95% Confidence Interval of the Difference |
|  |  | Upper |
|  | Equal variances assumed | -32.863441 |
|  | Equal variances not assumed | -32.597104 |

| **TEI Wnt4 protein content** (n=8) | | | **UC Wnt4 protein content** (n=8) | | |
| --- | --- | --- | --- | --- | --- |
| **Mean** | **SD** | **Std. Error Mean** | **Mean** | **SD** | **Std. Error Mean** |
| 84.477 | 7.636 | 2.699 | 49.342 | 13.170 | 4.656 |

| Tests of Normality | | | | | | |
| --- | --- | --- | --- | --- | --- | --- |
|  | Kolmogorov-Smirnov^a^ | | | Shapiro-Wilk | | |
|  | Statistic | df | Sig. | Statistic | df | Sig. |
| **TEI** | 0.331 | 3 | 0.000 | 0.865 | 3 | 0.281 |
| **UC** | 0.364 | 3 | 0.000 | 0.800 | 3 | 0.114 |

a. Lilliefors Significance Correction

| Independent Samples Test | | | | |
| --- | --- | --- | --- | --- |
|  | | Levene's Test for Equality of Variances | | t-test for Equality of Means |
|  |  | F | Sig. | t |
|  |  |  |  |  |
|  | Equal variances assumed | 1.051 | **0.323** | 6.528 |
|  | Equal variances not assumed |  |  | 6.528 |

|  | | | | |
| --- | --- | --- | --- | --- |
|  | | t-test for Equality of Means | | |
|  |  | df | Sig. (2-tailed) | Mean Difference |
|  |  |  |  |  |
|  | Equal variances assumed | 14 | **0.000** | 35.135758 |
|  | Equal variances not assumed | 11.228 | 0.000 | 35.135758 |

|  | | | |
| --- | --- | --- | --- |
|  | | t-test for Equality of Means | |
|  |  | Std. Error Difference | 95% Confidence Interval of the Difference |
|  |  |  | Lower |
|  | Equal variances assumed | 5.382340 | 23.591786 |
|  | Equal variances not assumed | 5.382340 | 23.318597 |

|  | | |
| --- | --- | --- |
|  | | t-test for Equality of Means |
|  |  | 95% Confidence Interval of the Difference |
|  |  | Upper |
|  | Equal variances assumed | 46.679730 |
|  | Equal variances not assumed | 46.952920 |

**Figure 1D.** miR27b expression levels of exosomes

| **RNA levels of control exosomes** (n=3) | | | **RNA levels of Wnt4 exosomes** (n=3) | | |
| --- | --- | --- | --- | --- | --- |
| **Mean** | **SD** | **Std. Error Mean** | **Mean** | **SD** | **Std. Error Mean** |
| 0.0356 | 0.0026 | 0.0015 | 0.2755 | 0.0076 | 0.0044 |

| Tests of Normality | | | | | | |
| --- | --- | --- | --- | --- | --- | --- |
|  | Kolmogorov-Smirnov^a^ | | | Shapiro-Wilk | | |
|  | Statistic | df | Sig. | Statistic | df | Sig. |
| **Ctrl exosomes** | 0.178 | 3 | 0.000 | 1.000 | 3 | 0.960 |
| **Wnt4 exosomes** | 0.176 | 3 | 0.000 | 1.000 | 3 | 0.985 |

a. Lilliefors Significance Correction

| Independent Samples Test | | | | |
| --- | --- | --- | --- | --- |
|  | | Levene's Test for Equality of Variances | | t-test for Equality of Means |
|  |  | F | Sig. | t |
|  |  |  |  |  |
|  | Equal variances assumed | 1.605 | **0.274** | -51.528 |
|  | Equal variances not assumed |  |  | -51.528 |

|  | | | | |
| --- | --- | --- | --- | --- |
|  | | t-test for Equality of Means | | |
|  |  | df | Sig. (2-tailed) | Mean Difference |
|  |  |  |  |  |
|  | Equal variances assumed | 4 | **0.000** | -0.23995826 |
|  | Equal variances not assumed | 2.453 | 0.000 | -0.23995826 |

|  | | | | |
| --- | --- | --- | --- | --- |
|  | | t-test for Equality of Means | | |
|  |  | Std. Error Difference | 95% Confidence Interval of the Difference | |
|  |  |  | Lower | Upper |
|  | Equal variances assumed | 0.00465685 | -0.25288775 | -0.22702877 |
|  | Equal variances not assumed | 0.00465685 | -0.25683377 | -0.22308274 |

| **RNA levels of TEI exosomes** (n=3) | | | **RNA levels of UC exosomes** (n=3) | | |
| --- | --- | --- | --- | --- | --- |
| **Mean** | **SD** | **Std. Error Mean** | **Mean** | **SD** | **Std. Error Mean** |
| 0.4680 | 0.0453 | 0.0262 | 0.0734 | 0.0056 | 0.0032 |

| Tests of Normality | | | | | | |
| --- | --- | --- | --- | --- | --- | --- |
|  | Kolmogorov-Smirnov^a^ | | | Shapiro-Wilk | | |
|  | Statistic | df | Sig. | Statistic | df | Sig. |
| **TEI exosomes** | 0.180 | 3 | 0.000 | 0.999 | 3 | 0.947 |
| **UC exosomes** | 0.178 | 3 | 0.000 | 1.000 | 3 | 0.958 |

a. Lilliefors Significance Correction

| Independent Samples Test | | | | |
| --- | --- | --- | --- | --- |
|  | | Levene's Test for Equality of Variances | | t-test for Equality of Means |
|  |  | F | Sig. | t |
|  |  |  |  |  |
|  | Equal variances assumed | 3.339 | **0.142** | 14.952 |
|  | Equal variances not assumed |  |  | 14.952 |

|  | | | | |
| --- | --- | --- | --- | --- |
|  | | t-test for Equality of Means | | |
|  |  | df | Sig. (2-tailed) | Mean Difference |
|  |  |  |  |  |
|  | Equal variances assumed | 4 | **0.000** | 0.39453767 |
|  | Equal variances not assumed | 2.061 | 0.004 | 0.39453767 |

|  | | | | |
| --- | --- | --- | --- | --- |
|  | | t-test for Equality of Means | | |
|  |  | Std. Error Difference | 95% Confidence Interval of the Difference | |
|  |  |  | Lower | Upper |
|  | Equal variances assumed | 0.02638701 | 0.32127559 | 0.46779974 |
|  | Equal variances not assumed | 0.02638701 | 0.28415689 | 0.50491844 |

**Figure 2-1.** TEC gene expression values by SYBR-green qPCR

| **DX** (n=3) | | | | | | | | | | | | | | | | | |
| --- | --- | --- | --- | --- | --- | --- | --- | --- | --- | --- | --- | --- | --- | --- | --- | --- | --- |
| **FoxN1** | | | **PPARγ** | | | **E-cadherin** | | | | **N-cadherin** | | | | **CIITA** | | | |
| **Mean** | **SD** | | **Mean** | | **SD** | **Mean** | | **SD** | | **Mean** | | **SD** | | **Mean** | | **SD** | |
| 0.247 | 0.189 | | 15.455 | | 10.636 | 0.159 | | 0.134 | | 9.256 | | 3.306 | | 0.248 | | 0.191 | |
|  | | | | | | | | | | | | | | | | | |
| **DX + Wnt4 exosomes** (n=3) | | | | | | | | | | | | | | | | | |
| **FoxN1** | | | | **PPARγ** | | | **E-cadherin** | | | | **N-cadherin** | | | | **CIITA** | | |
| **Mean** | | **SD** | | **Mean** | **SD** | | **Mean** | | **SD** | | **Mean** | | **SD** | | **Mean** | | **SD** |
| 16.79 | | 3.351 | | 1.321 | 1.803 | | 5.707 | | 0.0721 | | 0.825 | | 0.03 | | 6.869 | | 1.618 |

| **Figure 2-1.** Independent Samples Tests | | | | | | |
| --- | --- | --- | --- | --- | --- | --- |
|  | | | Levene's Test for Equality of Variances | | t-test for Equality of Means | |
|  |  |  | F | Sig. | t | df |
|  |  |  |  |  |  |  |
| **FoxN1** |  | Equal variances assumed | 1831.643 | **0.000** | -9.337 | 3 |
|  |  | Equal variances not assumed |  |  | -6.974 | 1.004 |

|  | | | | | |
| --- | --- | --- | --- | --- | --- |
|  | | | t-test for Equality of Means | | |
|  |  |  | Sig. (2-tailed) | Mean Difference | Std. Error Difference |
|  |  |  |  |  |  |
| **FoxN1** |  | Equal variances assumed | 0.003 | -16.542167 | 1.771755 |
|  |  | Equal variances not assumed | **0.090** | -16.542167 | 2.372022 |

|  | | | | | | |
| --- | --- | --- | --- | --- | --- | --- |
|  | | | Levene's Test for Equality of Variances | | t-test for Equality of Means | |
|  |  |  | F | Sig. | t | df |
|  |  |  |  |  |  |  |
| **PPAR gamma** |  | Equal variances assumed | 6.272 | **0.066** | 2.269 | 4 |
|  |  | Equal variances not assumed |  |  | 2.269 | 2.115 |

|  | | | | | |
| --- | --- | --- | --- | --- | --- |
|  | | | t-test for Equality of Means | | |
|  |  |  | Sig. (2-tailed) | Mean Difference | Std. Error Difference |
|  |  |  |  |  |  |
| **PPAR gamma** |  | Equal variances assumed | **0.086** | 14.134000 | 6.228313 |
|  |  | Equal variances not assumed | 0.144 | 14.134000 | 6.228313 |

|  | | | | | |
| --- | --- | --- | --- | --- | --- |
|  | | Levene's Test for Equality of Variances | | t-test for Equality of Means | |
|  |  | F | Sig. | t | df |
|  |  |  |  |  |  |
| **E-cadherin** | Equal variances assumed | 0.647 | **0.480** | -51.934 | 3 |
|  | Equal variances not assumed |  |  | -59.889 | 2.988 |

|  | | | | |
| --- | --- | --- | --- | --- |
|  | | t-test for Equality of Means | | |
|  |  | Sig. (2-tailed) | Mean Difference | Std. Error Difference |
|  |  |  |  |  |
| **E-cadherin** | Equal variances assumed | **0.000** | -5.547333 | 0.106816 |
|  | Equal variances not assumed | 0.000 | -5.547333 | 0.092627 |

|  | | | | | |
| --- | --- | --- | --- | --- | --- |
|  | | Levene's Test for Equality of Variances | | t-test for Equality of Means | |
|  |  | F | Sig. | t | df |
|  |  |  |  |  |  |
| **N-cadherin** | Equal variances assumed | 8.141 | **0.065** | 3.421 | 3 |
|  | Equal variances not assumed |  |  | 4.417 | 2.001 |

|  | | | | | |
| --- | --- | --- | --- | --- | --- |
|  | | t-test for Equality of Means | | | |
|  |  | Sig. (2-tailed) | Mean Difference | Std. Error Difference | 95% Confidence Interval of the Difference |
|  |  |  |  |  | Lower |
| **N-cadherin** | Equal variances assumed | **0.042** | 8.431833 | 2.464564 | 0.588491 |
|  | Equal variances not assumed | 0.048 | 8.431833 | 1.909124 | 0.219533 |

|  | | | | | |
| --- | --- | --- | --- | --- | --- |
|  | | Levene's Test for Equality of Variances | | t-test for Equality of Means | |
|  |  | F | Sig. | t | df |
|  |  |  |  |  |  |
| **CIITA** | Equal variances assumed | 394.896 | **0.000** | -7.658 | 3 |
|  | Equal variances not assumed |  |  | -5.760 | 1.019 |

|  | | | | | |
| --- | --- | --- | --- | --- | --- |
|  | | t-test for Equality of Means | | | |
|  |  | Sig. (2-tailed) | Mean Difference | Std. Error Difference | 95% Confidence Interval of the Difference |
|  |  |  |  |  | Lower |
| **CIITA** | Equal variances assumed | 0.005 | -6.620333 | 0.864531 | -9.371657 |
|  | Equal variances not assumed | **0.106** | -6.620333 | 1.149321 | -20.605619 |

**Figure 2-2.** SYBR-green qPCR primer sequences

| **Name of gene** | **Mouse primer sequence** |
| --- | --- |
| Actin-for | GGGAGGGTGAGGGACTTCC |
| Actin-rev | TGGGCGCTTTTGACTCAGGA |
| HPRT-for | TTGCTCGAGATGTCATGAAGGA |
| HPRT-rev | ATGTAATCCAGCAGGTCAGCA |
| FoxN1-for | AGGTCAAGGTCAAGCCCCAAG |
| FoxN1-rev | CGAACAGAATTCTTCCAGCCA |
| CIITA-for | TGATGGATGTCCAGTTCAACAAG |
| CIITA-rev | TGAAGGCTGGAGGCCAGTT |
| PPARg-for | TGTCTCACAATGCCATCAGGT |
| PPARg-rev | TCTTTCCTGTCAAGATCGCCC |
| E-Cadherin-for | AAGTGACCGATGATGATGCC |
| E-Cadherin-rev | CTTCATTCACGTCTACCACGT |
| N-Cadherin-for | GTGGAGGCTTCTGGTGAAAT |
| N-Cadherin-rev | CTGCTGGCTCGCTGCTT |

**Figure 3C.** Integrated pixel density values of mouse thymic sections

|  | **FITC** (n=5) | | **DiI** (n=5) | |
| --- | --- | --- | --- | --- |
| **Age** | **Mean** | **SD** | **Mean** | **SD** |
| 2-month-old | 1532538750 | 83111820.356 | 1065261000 | 35664176.242 |
| 21-month-old | 1328268400 | 170102611.121 | 1428699600 | 169016006.133 |

| **Figure 3C.** Tests of Normality | | | | | | |
| --- | --- | --- | --- | --- | --- | --- |
|  | Kolmogorov-Smirnov^a^ | | | Shapiro-Wilk | | |
|  | Statistic | df | Sig. | Statistic | df | Sig. |
| **FITC** | 0.183 | 8 | 0.200^*^ | 0.919 | 8 | 0.421 |
| **DiI** | 0.170 | 8 | 0.200^*^ | 0.937 | 8 | 0.583 |

*. This is a lower bound of the true significance.

a. Lilliefors Significance Correction

| **Figure 3C.** Independent Samples Test | | | | | |
| --- | --- | --- | --- | --- | --- |
|  | | Levene's Test for Equality of Variances | | t-test for Equality of Means | |
|  |  | F | Sig. | t | df |
|  |  |  |  |  |  |
| **FITC** | Equal variances assumed | 0.726 | **0.422** | 2.181 | 7 |
|  | Equal variances not assumed |  |  | 2.357 | 6.028 |

|  | | | | | |
| --- | --- | --- | --- | --- | --- |
|  | | t-test for Equality of Means | | | |
|  |  | Sig. (2-tailed) | Mean Difference | Std. Error Difference | 95% Confidence Interval of the Difference |
|  |  |  |  |  | Lower |
| **FITC** | Equal variances assumed | **0.066** | 204270350.00 | 93662038.95 | -17205178.75 |
|  | Equal variances not assumed | 0.056 | 204270350.00 | 86682601.10 | -7597541.14 |

|  | | | | | |
| --- | --- | --- | --- | --- | --- |
|  | | Levene's Test for Equality of Variances | | t-test for Equality of Means | |
|  |  | F | Sig. | t | df |
|  |  |  |  |  |  |
| **DiI** | Equal variances assumed | 2.290 | **0.181** | -3.567 | 6 |
|  | Equal variances not assumed |  |  | -4.639 | 4.565 |

|  | | | | | |
| --- | --- | --- | --- | --- | --- |
|  | | t-test for Equality of Means | | | |
|  |  | Sig. (2-tailed) | Mean Difference | Std. Error Difference | 95% Confidence Interval of the Difference |
|  |  |  |  |  | Lower |
| **DiI** | Equal variances assumed | **0.012** | -363438600.00 | 101897337.56 | -612772402.86 |
|  | Equal variances not assumed | 0.007 | -363438600.00 | 78340665.61 | -570724329.30 |

**Figure 4B.** DiI expression values of mouse organs (pilot study)

|  | **Total radiant efficiency**  [p/s] / [µW/cm^2^] | |  |
| --- | --- | --- | --- |
| **Organs** | **Wnt5a mouse** (n=1) | **Wnt4 mouse** (n=1) |  |
| **Thymus** | **5.28 x 10^8^** | **3.74 x 10^8^** |  |
| **Liver** | **3.61 x 10^10^** | **1.91 x 10^10^** |  |
| Lung | 1.07 x 10^9^ | 4.96 x 10^8^ |  |
| Spleen | 2.8 x 10^8^ | 1.71 x 10^8^ |  |
| **Thymus relative to liver** | **0.01462** | **0.01958** |  |

|  |
| --- |

**Figure 5C.** Integrated pixel density values of mouse thymic sections (pilot study)

|  | **FITC** | **DiI** |
| --- | --- | --- |
| **Mouse** | **Int. density** | **Int. density** |
| **Ctrl**  (n=1) | 8.99 x 10^8^ | 0 |
| **Wnt4 exosomes**  (n=1) | 8.88 x 10^8^ | 1.23 x 10^9^ |
